# Dispersion and Performance of a Nanoclay/Whey Protein Isolate Coating upon its Upscaling as a Novel Ready-to-Use Formulation for Packaging Converters

**DOI:** 10.3390/polym11091410

**Published:** 2019-08-28

**Authors:** Elodie Bugnicourt, Nicola Brzoska, Esra Kucukpinar, Severine Philippe, Enrico Forlin, Alvise Bianchin, Markus Schmid

**Affiliations:** 1 IRIS Technology Solutions S.L., Parc Mediterrani de la Tecnologia, 08860 Barcelona, Spain; 2 Fraunhofer-Institute for Process Engineering and Packaging IVV, 85354 Freising, Germany; 3 Visum, NexusUCD, University College Dublin, D04 V1W8 Dublin, Ireland; 4 MBN Nanomaterialia S.p.A., 31050 Vascon di Carbonera (TV), Italy; 5 Faculty of Life Sciences, Albstadt-Sigmaringen University, 72488 Sigmaringen, Germany

**Keywords:** dispersion, coatings, whey protein isolate, nanocomposites, nanoclay, barrier, morphology

## Abstract

Studies on composition optimisation showed that the mixing of nanoclays to whey protein-isolate (WPI)-based coating formulations offers an effective strategy to reduce the oxygen permeability of coated polymer films. The scaling up of the various processing stages of these formulations was undertaken to prove their industrial feasibility. The aim was to investigate the effect of various preparation methods at different production scales (pilot- and semi-industrial scale) on the barrier performance and morphological properties of the applied nanocomposites. A nano-enhanced composition was converted into a so-called “ready-to-use” formulation by means of a solid-state pre-dispersion process using ball-milling. The process yielded a nearly dust-free, free-flowing powder containing agglomerated particles, which can easily be mixed with water. The preparation of a coating formulation using the ready-to-use granules and its upscaling for roll-to-roll converting at pilot- and semi-industrial scale was also successfully implemented. The effects of both the production at various scales and ultrasound treatment on the morphology and barrier performance of the nanocomposites were characterized by transmission electron microscopy, scanning electron microscopy, as well as oxygen permeability measurements. Results have shown that the addition of nanoclays to WPI-based coating formulations ultimately led to significantly reduced oxygen permeabilities to 0.59 cm^3^, 100 µm·m^−2^·d^−1^·bar^−1^ (barrier improvement factor, BIF of 5.4) and 0.62 cm^3^, 100 µm·m^−2^·d^−1^·bar^−1^ (BIF of 5.1) in cases of pilot- and semi-industrial-processed coatings, respectively, compared to a reference without nanoclay. In both cases, a similar degree of nanoparticle orientation was achieved. It was concluded that the solid state pre-dispersion of the nanoplatelets during the production of the ready-to-use formulation is the predominant process determining the ultimate degree of nanoparticle orientation and dispersion state.

## 1. Introduction

The ability of protein-based films and coatings to act as a superior barrier against the permeation of oxygen in packaging materials has attracted a great deal of attention in recent years, as extensive research has demonstrated [1,2,3,4,5,6,7,8,9,10,11]. Research has shown that the application of coating formulations based on native whey proteins followed by in-line denaturation, in contrast to the application of preliminary denatured whey proteins formulations, allows the processing of formulations with much higher solid contents while still providing an optimum barrier against oxygen [12,13]. 

While currently being currently routed to commercialisation, whey protein-based coatings offer several advantages compared to conventional petrochemically-sourced oxygen barrier materials, such as poly (ethylene–*co*–vinyl alcohol) (EVOH), as they are extracted from renewable resources and, despite usually being biodegradable, also offer the opportunity to be recycled [14]. Indeed, upon the enzymatic removal of whey protein-based coating layers from multilayer films and laminate, these coatings can contribute to end-of-life product management as a sub sequential film, and laminate delamination allows for the separate recycling of the materials [15]. Moreover, when applied on biodegradable polymeric substrates, e.g., poly (lactic acid) (PLA), the design and production of compostable packaging concepts is possible [16]. These aspects enable converters in the plastics packaging sector to cope with the EU action plan for a circular economy, which was adopted by the European commission in 2015 and supports the goal that all plastic packaging is recyclable by 2030 [17]. 

However, protein-based films still have drawbacks that need to be addressed in order to allow them to be competitive with conventional established materials in the market place. This particularly includes the excellent but still higher oxygen permeability of neat whey protein isolate (WPI) in comparison to petroleum-based high barrier materials, such as poly (ethylene–*co*–vinyl alcohol) (EVOH) or copolymers of poly (vinylidene chloride) [18]. 

One effective strategy to address this drawback is the application of nanocomposites [19,20,21,22]. Among other biopolymers, nanocomposites based on whey proteins have become a promising field of research [23,24,25,26,27,28]. Amongst other researchers, we have studied nanocomposite coatings for their ability to improve the barrier performance of these coatings by extending the pathway and time and reducing the effective volume fraction for diffusing molecules, which is generally referred to as the tortuous-path effect [29]. In order to fully take advantage of these effects, the uniform and nanoscale dispersion of the nanoparticles is required [30]. In the case of layered silicates, the formation of a nanocomposite depends on several factors, such as the nature and type of the matrix, their compatibility, as well as the dispersion process [31]. True nanocomposites with enhanced properties comprise an intercalated or exfoliated structure, in contrast to phase-separated micro composites, which do not achieve enhanced barrier properties [31,32]. Previously published studies have compared different coating formulations and been dedicated to selecting a suitable plasticiser and nanoparticle type and concentration necessary to obtain optimized oxygen barrier performance with an emphasis on the processability of the nano-enhanced formulations [33,34]. 

The present study goes one step further than the prior art in the upscaling of nano-enhanced whey protein-based coating formulations and focuses on the influence of the dispersion process of a ready-to-use coating formulation on the final oxygen barrier performance and morphology of nanocomposite coatings processed at different scales. 

## 2. Materials and Methods

### 2.1. Materials

WPI was obtained from Agropur Ingredients (Saint-Hubert, Laval, QC, Canada; previously Davisco Food International Inc.). Aqueous nanoclay dispersions were provided by ITENE, Valencia, Spain, as described in our previously published results [34]. The plasticising agent, as selected in a prior published study that focused on formulation optimization [34], was purchased from Panreac S.A., Barcelona, Spain to be used as a plasticiser for the WPI-based film-forming formulations. As a substrate for the coatings, chemically pre-treated poly (ethylene terephthalate) (PET) with thicknesses of 23 and 100 µm, respectively (PLASTIKA-ANDREJ MESOJEDEC S.P., Polje, Slovenia), were respectively used for the application of coating formulations using a pilot-scale and a semi-industrial-scale processing environment. 

#### 2.1.1. Preparation of WPI-Based Ready-to-Use Formulations by High-Energy Ball-Milling

WPI was mixed, using a high-energy ball-milling approach, with plasticizer and nanoclay according to a coating formulation that was selected based on previously published results [33,34]. Dry ball-milling of the formulation constituents in form of a fine powder (WPI, plasticizer and nanoclay) was performed by a proprietary process of MBN, specifically adapted for the manufacturing of ready-to-use formulations for biopolymer nanocomposites. The homogeneous dispersion of formulation constituents as well as an exfoliation effect of the utilized nanoclay was achieved in dry conditions, without the addition of any solvent. The fine and light-weighted powder obtained was then agglomerated in form of granules by using a minimum amount of water, which highly reduced the dustiness of the mixture. The ready-to-use formulation, which is in form of granules, just needs to be mixed with water, as described below, for obtaining the feedstock dispersion for coating processes. In order to be able to interpret the results achieved with the nano-enhanced WPI-based coating formulation, a pristine ready-to-use formulation was also milled and agglomerated without the addition of nanoclay. 

#### 2.1.2. Coating Preparation, Application, and Drying

Coating formulations for pilot- and semi-industrial-scale processing with and without nanoparticles were obtained by mixing agglomerated ready-to-use formulations with deionized water. In contrast to pilot- and semi-industrial-scale, coating formulations based on pre-dispersed ready-to-use granules were not prepared for lab-scale coatings. Instead, the formulation constituents were mixed and the aqueous nanodispersions were dispersed altogether. The solid content was kept at about 18.2% (*w*/*w*, referred to the total weight of the aqueous formulation) for all coating formulations. The nanoparticle concentration was at about 15% (*w*/*w*, relative to the WPI content of the ready-to-use formulation [34]). This corresponds to a final nanoparticle concentration of approximately 9% (*w*/*w*, relative to the dry coating weight of the WPI/plasticiser solids). 

##### Lab-Scale

For lab-scale processing, aqueous protein-plasticizers formulations with (in that case using the above mentioned clay dispersions from ITENE) and without nanoparticles were mixed at 23 °C and 130 rpm in a magnetic stirrer for 24 h. 

In order to understand the effect of an ultrasonication treatment on the intercalation/exfoliation state of the used nanoclay, some of the coating formulations were treated with an ultrasound horn for up to 240 min at 20 µm of amplitude. Treatment by ultrasound was realized by recirculation of the prepared sample. While the treatment with ultrasound was taking place, the sample was agitated with a magnetic stirrer at 100 rpm. The ultrasonic system was from ISM-Industrial Sonomechanics (Miami, FL, USA) and included a 1200 W ultrasonic generator, piezoelectric transducer, a full-wave Barbell Horn, and an 80 mL reactor chamber with cooling jacket. Subsequently, about 5 mL of these still native coating formulations were then applied on a 100 µm chemically pre-treated PET film using a wired-rod (Lumaquin, Barcelona, Spain, 100 µm wet-film thickness).

In order to understand the effect of an ultrasonication treatment on the intercalation/exfoliation state of the used nanoclay, samples coated with and without ultrasound-treated formulations have been compared in terms of their dispersion quality and gas barrier performance. For this purpose, a nanodispersion provided by ITENE (Valencia, Spain) was mixed with a WPI-based aqueous formulation and treated with an ultrasound horn for variable times of up to 240 min at 20 µm of amplitude. Samples were taken at different time-intervals. Treatment by ultrasound was realized by recirculation of the prepared sample. While the treatment with ultrasound was taking place, the sample was agitated with a magnetic stirrer at 100 rpm.

The coatings were dried in a stove at (50 ± 2) °C for 5 min and then kept at ambient conditions (57% RH and 20.7 °C) for at least 15 h. Each coating was carried out in four replicas. Although the drying procedure is far from the optimal that induces in-process denaturation to maximise oxygen barrier properties, it allows one to obtain comparative results to show the effect of nanoparticles. 

##### Pilot-Scale

For pilot-line processing, the aqueous coating formulations obtained using the ready-to-use granules were mixed at 23 °C and 200 rpm in an electrically heatable stirrer (Thermomix 31-1, Vorwerk Deutschland Stiftung & Co. KG, Berlin, Germany) for 30 min, and then at 90 °C and 400 rpm for another 30 min. The heat-treatment induces the denaturation of the native whey proteins. Subsequently, both formulations, with and without nanoparticles, were transferred into a 2.5 L glass bottle and cooled down to room temperature using a water bath and an electrical stirrer at 200 rpm. Both formulations were degassed in an ultrasonic bath (DT 514 H, Ultrasonic peak output: 860 W, bandelin electronic GmbH & Co. KG, Berlin, Germany) at 25 °C and 37 kHz for 30 min to allow incorporated air to escape.

Pre-denatured WPI-based coating formulations were applied on a 23 µm chemically pre-treated PET film using a reverse-gravure coating system by a pilot-scale lacquering and lamination line (available at Fraunhofer IVV, Freising, Germany) at width of 360 mm. The ceramic-coated anilox roller had a helically-engraved (Hachure) surface with 14 lines per centimeter and an angle of 45° diagonally to the main axis, resulting in a theoretical wet-film coating volume of approximately 90 mL·m^−2^. The web-speed during the coating application was 5 m·min^−1^, whereas the rotational speed of the gravure-roll was 6 m·min^−1^. 

The coatings were dried convectively using hot air at a temperature of 120 °C in an in-line drying-tunnel with a dwell-time of approximately 90 s. For both coating formulations, with and without nanoparticles, single-, double-, and triple-layer coatings were performed at dry layer thicknesses of approximately 10, 17, and 30 µm, respectively. This leads to a multiple drying of the first and the second-layer coatings. 

##### Semi-Industrial-Scale

For the semi-industrial processing-line, the aqueous coating formulations obtained using the ready-to-use granules were mixed at 23 °C and 2 rpm for 30 min with a three-bladed propeller stirrer (diameter 10 cm). The still native WPI-based coating dispersions were then put to rest over night to allow incorporated air to escape. Prior to coating application, a quantity of the aqueous nano-enhanced formulation was exposed to an ultrasonication-treatment (1200 W, 20 µm amplitude) similar to the lab-scale trials with the aim to investigate a possible effect on the barrier performance and layer morphology of the resulting coatings. However, in order to avoid possible re-agglomeration of the particles that were already exfoliated during solid-state pre-dispersion of the ready-to-use formulation, its exposure was limited to a 1 min time-interval. 

Native WPI-based coating formulations were applied on a 100 µm chemically pre-treated PET film using an engraved application roller on a semi-industrial coating machine (available at the company Lajovic Tuba d.o.o., home built with support from IRIS Technology Solutions, Barcelona, Spain). 

The coatings were dried using a specifically built convective and infrared drying section, which provides a finely adjustable temperature profile, following previously published research results [12] and patented conditions [13]. These conditions ensure an optimal in-process denaturation of the native whey protein fraction of the coating formulation. Further visual understanding of the in-line drying process is available in the supplementary materials section. 

### 2.2. Methods

#### 2.2.1. Transmission Electron Microscopy

To ascertain the need for a sonication of the liquid coating formulations, transmission electron microscopy (TEM) was performed on selected samples coated in lab scale at the UCD Conway Institute of Biomolecular and Biomedical Research (University College Dublin, Dublin, Ireland), using protocols developed previously for delicate biological samples to avoid degrading the biopolymer coatings, whereby fixation is performed in two steps, first by glutaraldehyde fumes, then by Osmium fumes. The sample is then plunged into acetone, followed by a multi-step substitution by an epoxy resin, and then a final polymerization step is carried out. 

#### 2.2.2. Scanning Electron Microscopy

To determine the layer thicknesses of the coated samples and the intercalation/exfoliation state of the nanoparticles within the WPI-based matrix, specimens taken from pilot and semi-industrial scale produced coatings were prepared for scanning electron microscopy (SEM) investigations. Specimens were cut into small pieces and adhered between two silicon-wafers using a conductive two-component-adhesive and were then cured in a vise to maintain a minimal pressure for at least 30 min at ambient conditions. The cross-sectional interfaces were prepared by an Ar^+^-ion beam, using a cross-section polisher IB-19530CP (JOEL Ltd., Akishima, Japan). Samples were coated with a thin gold layer to reduce electrical charging of the non-conducting polymeric layers. SEM images were taken using a SEM JSM-7200F (JOEL Ltd., Akishima, Japan) at various accelerating voltages and magnifications. Layer thicknesses of the investigated samples could be determined using a software package (JOEL Ltd., Akishima, Japan). 

#### 2.2.3. Oxygen permeability

The coulometric oxygen permeability (OP) measurements were performed at 23 °C and 50% RH using an Ox-Tran^®^ 2/20 measurement device from Mocon, Brooklyn Park, MN, USA according to DIN 53380-3. A two-fold determination was performed in all cases. The oxygen permeability values, *Q*_coating_, for the applied coatings were calculated according to

(1) $$\frac{1}{Q_{\mathrm{coating}}}=\frac{1}{Q_{\mathrm{total}}}-\frac{1}{Q_{\mathrm{PET}}}$$

where *Q*_total_ is the oxygen permeability measured for the coated PET substrate. *Q*_PET_ is the oxygen permeability of the PET, and it is measured as 48.7 and 12.5 m^3^·m^−2^·d^−1^·bar^−1^ at 23 °C and 50% RH for the PET layer thicknesses of 23 and 100 µm, respectively. In order to allow direct comparisons between the different coatings independent of the coating layer thicknesses, the oxygen permeability, *Q*_coating_, was calculated for a layer thickness of 100 µm (*Q*_100_) by

(2) $$Q_{100}=Q\times \frac{d}{100}$$

where *d* is the dry coating layer thickness. 

## 3. Results

### 3.1. Effect of Ultrasound-Treatment on Lab-Scale Coating Quality Prepared from Liquid Nanoparticles Suspensions

At lab scale, nanocomposite coatings of an initially native nano-enhanced formulation were produced with the aim to determine the effect of an additional ultrasound-treatment on the dispersion quality of the aqueous WPI-based coating formulation. As a reference, a formulation produced with the exact same coating process parameters, but without nanoparticles, was prepared as previously described (Lab-Scale). 

**Figure 1:**
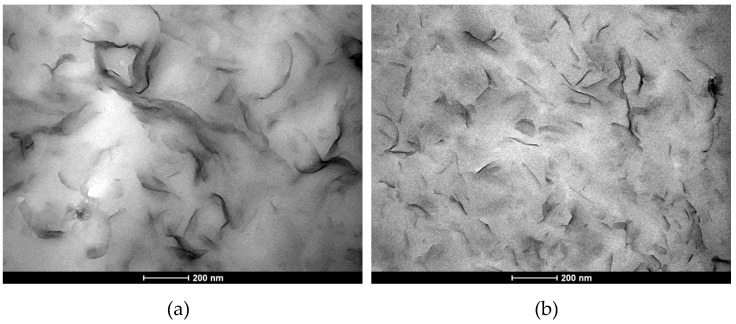
Transmission electron microscopy (TEM) image of coated samples produced at lab scale (6000× magnification) (**a**) without ultrasonication-treatment and (**b**) after 240 min of ultrasonication-treatment at 20 µm amplitude.

Transmission electron microscopy (TEM) was used to analyse the layer morphology of untreated coatings and coatings, which were treated with ultrasonication for 240 min (Figure 1). For coatings that were treated by ultrasonication, it seems that fewer nanoparticle agglomerates were present in the dry coatings, and more individual nanoplatelets, instead of nanoplatelet-stacks, achieved an intercalated or exfoliated state (Figure 1b). 

Nevertheless, as TEM micrographs cannot lead to a quantitative conclusion, the study was complemented by X-ray diffraction and oxygen permeability measurements. X-ray diffraction measurements on cast-film samples obtained from the same coating formulations did not reveal any relevant diffraction peak, confirming the absence of short distance organisation also observed by the TEM micrographs. There is no difference in the oxygen permeability values measured for the coated specimens produced with and without using ultrasonication treatment, despite a more homogeneous distribution of the nanoparticles (Figure 1b).

### 3.2. Oxygen Barrier Performance of Coatings Based on Ready-to-Use Formulations

**Figure 2:**
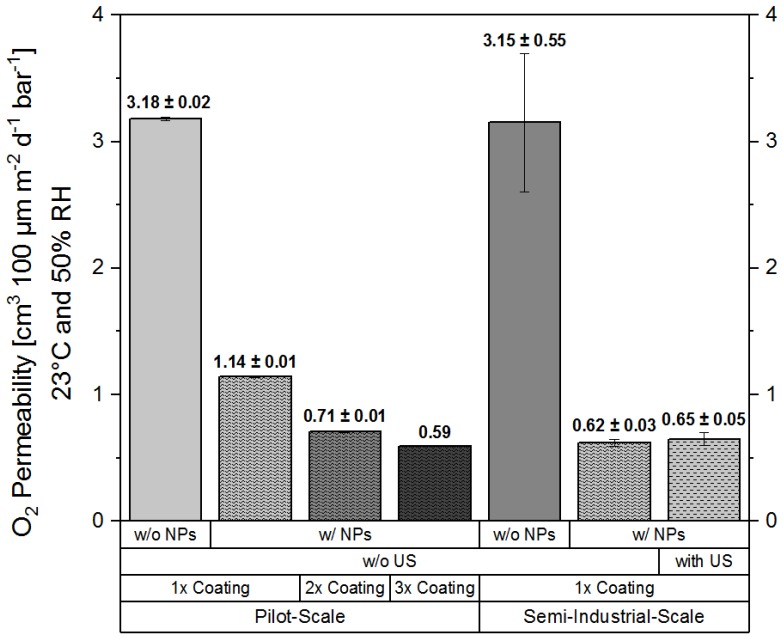
Oxygen permeability (*Q*_100_ at 23 °C and 50% RH of WPI-based coatings calculated for a coating layer thickness of 100 µm. Effects of nanoparticle (NP) addition, number of coating/drying steps (1×, 2×, and 3× coating), ultrasound treatment (US, with and without (w/o)), and manufacturing environment (pilot-, semi-industrial-scale) on the WPI-based coating formulations prepared using novel ready-to-use agglomerates are shown. The data shown represent mean values of a two-fold measurement ± minimum/maximum deviation (range of variation).

Oxygen barrier performance measurements were performed for WPI-based nanocomposite coatings processed at pilot- and semi-industrial-scale at Fraunhofer-IVV and TUBA, respectively. Figure 2 shows the oxygen permeability (*Q*_100_) of the coating layer at 23 °C and 50% RH. The permeability values were calculated for a coating layer thickness of 100 µm according to Equations (1) and (2) in order to be able to compare the barrier performance of the various coatings with each other. 

Barrier performance measurements for the samples produced at semi-industrial scale have shown that for these coatings, an average oxygen barrier performance improvement of a factor of about 5.1 ((0.62 ± 0.03) cm^3^·m^−2^·d^−1^·bar^−1^ at 23 °C and 50% RH) could be achieved due to the addition of nanoclay, in comparison to pristine coatings. On the other hand, no significant difference in the oxygen barrier performance was observed for the coatings pre-treated by ultrasonication. The absence of a significant effect on the barrier performance due to ultrasound treatment might be explained by the efficiency of the solid-state pre-dispersion process. During the high-energy ball-milling process, resulting high shear-rates likely lead to the intercalation of polymeric protein chains between exfoliated nanoplatelets. Good compatibility between the surface of the studied nanoplatelets, owing to their partially polar surface, and the highly polar WPI-based matrix [33], as well as the ability to form interfacial interactions between nanofiller and WPI via hydrogen bonding [35], favor the dispersion of the particles and probably contribute to maintaining their dispersed state. It seems likely that upon reconstitution of the ready-to-use mix with water, the pre-dispersed nanoplatelets remain homogeneously dispersed within the WPI matrix, as they are stabilised by intermolecular interactions with protein side chains. 

At pilot-scale, coating trials were performed using the same nano-enhanced/pristine ready-to-use formulations, except that they were heat-treated prior to their coating on PET, as described in Section 3.2. The aim was to determine the effect of the number of coating/drying runs (single, double, or triple coating) on the oxygen permeability of the produced coatings. As expected, these coatings ultimately showed a very similar *Q*_100_ value of about 0.59 cm^3^·m^−2^·d^−1^·bar^−1^ at 23 °C and 50% RH, compared to the coatings produced at semi-industrial scale. However, it has to be noted here that the extent of barrier improvement in the case of pilot-scale processed coatings highly depended on the number of coating/drying runs. 

After application of a single coating layer of the nano-enhanced formulation, the oxygen permeability was reduced by a factor of about 2.8, in comparison to pristine WPI coatings, which had a *Q*_100_ value of about 3.2 cm^3^·m^−2^·d^−1^·bar^−1^ (Figure 2). By the application of the second coating layer, the *Q*_100_ value was reduced down to 0.7 cm^3^·m^−2^·d^−1^·bar^−1^, resulting in a barrier improvement factor of about 4.5. After the application of a third coating layer, the *Q*_100_ value was reduced to a value of about 0.59 cm^3^·m^−2^·d^−1^·bar^−1,^ which corresponds to a barrier improvement factor of about 5.4 in comparison to the permeability (*Q*_100_) of pristine WPI (Figure 2). 

The improvement of the barrier performance against oxygen permeation, which, in case of pilot-scale coatings, was only achieved after a second and third coating/drying step, can likely be attributed to a lower residual moisture content of the coatings due to longer corresponding drying times. High moisture contents lead to a swollen biopolymer matrix, as residual water molecules cluster between highly polar side chains of the hydrophilic WPI and plasticiser molecules, consequently increasing the free volume within the WPI matrix [34,36]. The significance of the residual water content with regard to the possible barrier improvement against oxygen permeation is also underlined by the fact that coatings produced at semi-industrial-scale, in contrast to pilot-scale, already result in low oxygen permeabilities after the first coating and drying step, which might be explained by a better drying performance of the combination or infra-red and convective air dryers at semi-industrial-scale, resulting in optimal in-line process denaturation conditions for the whey proteins [12,13]. 

Two other aspects need to be discussed regarding the oxygen barrier performance of the studied nanocomposite coating formulations. Firstly, the reduction of the effective volume available for the diffusing gas molecules due to the volume fraction that is occupied by the nanofiller and, secondly, an increase in the effective path length that the diffusing gas molecules, have to be covered due to the incorporated filler material [29]. 

**Figure 3:**
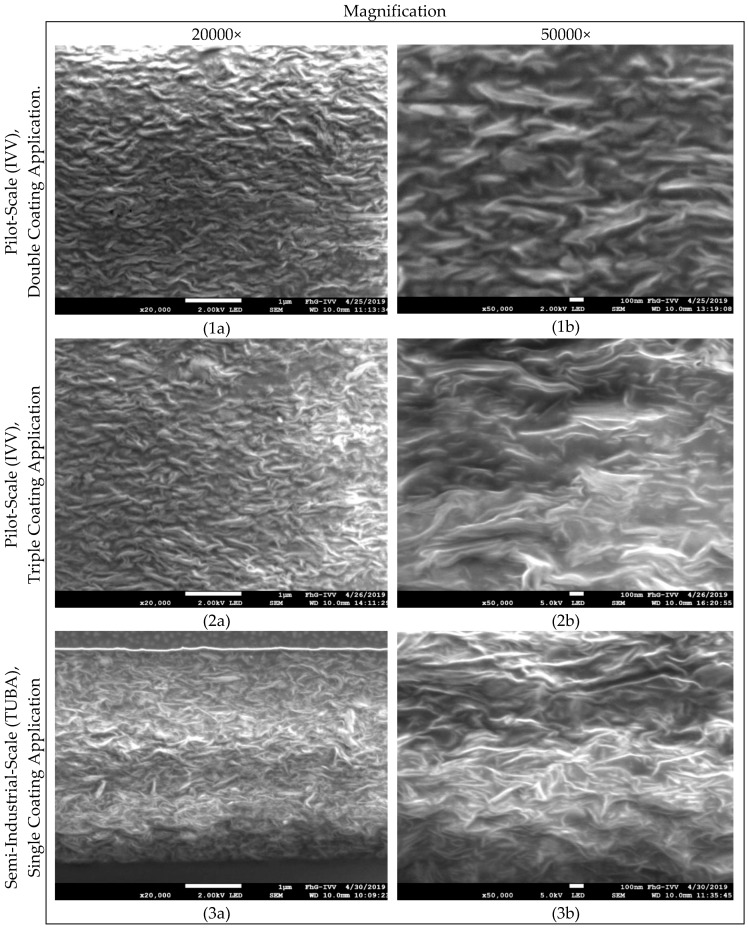
Scanning electron microscopy (SEM) images, at different magnifications, (**a**) at a magnification of 20,000 and (**b**) at a magnification of 50,000, of whey protein-isolate (WPI)-based nanocomposite coatings produced at both (1,2) pilot- and (3) semi-industrial-scale.

The effectiveness of the nanoparticles to significantly increase the diffusion path, the so-called tortuosity-effect, largely depends on the shape of the particles, where in an optimum case for barrier improvement platelet-shaped particles are oriented parallel to the plane of the film, and on their uniform distribution within the polymeric matrix. To prove the hypothesis of a lowered volume that is available for diffusion processes and a prolonged diffusion path due to nano-filler addition, the microstructure of the studied coatings has been examined by scanning electron microscopy (Figure 3). In Figure 3, Images 1a and 1b as well as 2a and 2b show specimen of coatings produced by double or triple coating at pilot-scale (IVV), respectively, whereas the images 3a and 3b show specimen of coatings produced at semi-industrial-scale (TUBA). Inorganic materials are perceived as lighter shades, whereas the organic polymer matrix as well as cavities are depicted in darker shades. 

Although few nanoparticle agglomerates are visible in all studied specimen, SEM images generally show a homogenous distribution of the added nano-filler within the WPI-based matrix. Based on these images, it can be concluded that in fact volume fraction available for the diffusing gas molecules was effectively reduced due to the volume fraction that is occupied by the nanofiller, and that the effective diffusion path for permeating gas molecules was significantly increased, which led to a reduction in oxygen permeability compared to pristine coatings without any nanofiller. However, despite their homogenous dispersion, no apparent differences regarding the orientation or intercalation/exfoliation state of the nanoparticles could be identified for specimens produced at semi-industrial and pilot-scale, either at a magnification of 20,000× (Images 1a, 2a, and 3a.) or at a higher magnification (50,000×) (Images 1b, 2b, and 3b). 

This indicates that despite the differences in the preparation method of the aqueous coating formulations at the different production scales, for the most part viscosity differences due to the sequence of the processing steps (since the viscosity of the WPI-based dispersions increases after heat-treatment due to denaturation-associated unfolding of proteins) as well as differences regarding the applied shear-rates (the ultimate degree of particle orientation and the intercalation/exfoliation state of the particles) are mainly affected by the solid state pre-dispersion of the nanoplatelets by means of high-energy ball-milling during the production of the ready-to-use formulation. 

## 4. Discussion

The present study highlights the potential of the studied ready-to-use coating formulations to be utilized at an industrial scale. Homogeneous and nano-scale dispersion of nanoparticles within the continuous (bio)polymer matrix is an essential feature in the formation of (bio-)nanocomposite coatings [30]. More precisely, in the case of layered silicates, to really take advantage of the addition of layered-silicate nano-fillers into a biopolymer matrix, an intercalated or exfoliated structure, which refers to an intermingling of the two phases, has to arise in order to significantly improve the properties of the resulting nanocomposites compared to traditional microcomposites, in which the two phases remain separated [20,31,32]. 

The successful implementation of the nano-enhanced, ready-to-use formulations prepared within this work demonstrates their applicability at different scales and processing conditions. However, it should be noted here that the application of a single coating in contrast to the necessity of multiple coating and drying steps to achieve the desired barrier performance and the in-process partial denaturation of the WPI, compared to time-consuming and costly pre-denaturation processes, is industrially far more feasible. 

A significantly reduced oxygen permeability of about 0.6 cm^3^, 100 µm·m^−2^·d^−1^·bar^−1^ in case of pilot- and semi-industrial-processed coatings represents an improvement factor against the permeation of oxygen of about 5.4 and 5.1, respectively, in comparison to the corresponding pristine reference coatings. The barrier performance against oxygen permeation of nano-enhanced coatings produced at pilot- or semi-industrial-scale seems to be independent of the shear-rates applied during the coating application with different coating units. 

A previous publication that studied the addition of layered-silicates into a WPI-based matrix reported a higher oxygen barrier improvement factor of up to 7.1 [34]—for coatings using the same nanofiller concentration as well as plasticiser type and concentration as in the present study, with an absolute oxygen permeability (*Q*_100_ coating monolayer) of about 0.6 cm^3^, 100 µm·m^−2^·d^−1^·bar^−1^. Whereas the absolute permeability value that was achieved by the addition of the nanofiller was the same as previously reported, the relative improvement factor was lower in the present study (factors 5.4 to 5.1), as the pristine reference coating (without any nanofiller-addition) already had a lower oxygen permeability due to the processing method (lab-scale [34] vs. pilot-scale (this study)) and drying procedure, as previously described (Lab-Scale and Pilot-Scale).

Moreover, it should be mentioned here that the used nano-filler material had an aspect ratio of about 70, as previously published by another study [37]. In the case of rectangular, platelet-shaped particles, the aspect ratio was between their diameter and thickness. Further optimization potential lies in the usage of nanoclays with higher aspect ratios, which will likely result in even more pronounced tortuosity effects at even lower nanofiller-loadings than already achieved within this work, as demonstrated in another study [33] with a different layered-silicate. 

In terms of their oxygen barrier performance, WPI-based nanocomposite coatings could therefore have the potential to eventually substitute commercially available petroleum-based oxygen barrier materials, such as certain grades of poly (ethylene–*co*–vinyl alcohol) with high ethylene contents (EVOH 44%) or certain halogen-containing poly (vinylidene chloride) copolymers, in multi-layer packaging applications. These high-performance materials exhibit oxygen permeabilities in the magnitude of about 0.24 to 0.11 cm^3^, 100 µm·m^−2^·d^−1^·bar^−1^, respectively. 

The process yielded a nearly dust-free, free-flowing powder containing agglomerated particles that was easily reconstituted with water. The preparation of a coating formulation using the ready-to-use granules and its upscaling for roll-to-roll converting at pilot- and semi-industrial scale was successfully implemented. The effects of production at various scales and of ultrasound treatment on morphology and barrier performance of the WPI-based nanocomposites were characterized by transmission electron microscopy, scanning electron microscopy, as well as oxygen permeability measurements. In both cases, a similar degree of nanoparticle orientation could be achieved. It was concluded that the solid state pre-dispersion of the nanoplatelets during production of the ready-to-use formulation is the predominant factor affecting the ultimate degree of nanoparticle orientation and dispersion state. Thereby, the most challenging process step for the upscaling of nanoclay/whey protein isolate coatings is the fabrication of the pre-dispersed “ready-to-use” granules. This process step facilitated a homogeneous distribution of nanoparticles within the final dry WPI matrix without the need of applying high shear-rates after mixing the “ready-to-use” granules with water prior to its coating. Several other factors, such as type of the coating application system, coating speed, or drying conditions for maintaining protein denaturation, as well as the rheology of the nano-enhanced formulations, still need to be considered during the different upscaling stages, as shown in previous publications [12,18,34].

However, one has to bear in mind that images obtained by SEM offer only very limited information due to the small quantity of material investigated and possible heterogeneities of the material. Additionally, it has to be noted that residual moisture within highly hygroscopic biopolymer matrices, at relevant pressures during the imaging process, evaporates, and can influence the microstructure of the material that is investigated. 

## 5. Conclusions

This study showed that the utilization of ready-to-use coating formulations in form of pre-dispersed agglomerates offers several advantages for flexible film converters in regard to the processability of nano-enhanced WPI-based coating formulations. 

Barrier performance measurements revealed that due to the presence of nanoclay within the biopolymer matrix, the oxygen permeability (*Q*_100_) of WPI-based nanocomposite coatings could be decreased significantly, in the case of coating formulations processed at both pilot- and semi-industrial scales. However, for coatings produced at pilot scale without a specifically adjusted drying process, the ultimate oxygen barrier performance highly depended on effective drying times, as shown by an increasing barrier performance after the second and third drying step.

The utilization of prolonged ultrasound treatment of the aqueous nano-enhanced coating formulations produced at lab scale demonstrated that it had an effect on the homogenous dispersion of larger nanoparticle agglomerates; however, no significant differences regarding the oxygen barrier performance were observed. This approach was shown to be less efficient than the use of a solid-state pre-dispersion, as this process is more feasible at an industrial scale and also does not require a further in-line ultrasonication process.

Images obtained by SEM indicated that the nanoparticle orientation and its state of intercalation/exfoliation in the case of both coatings produced at pilot- or semi-industrial-scale were quite similar. The obtained cross-sectional images of the nanocomposite coatings suggest that the effective diffusion path for permeating gas molecules was significantly increased and the available volume for diffusion processes was significantly reduced by the addition of the nano-filler into the polymeric matrix.

This leads to the conclusion that the ultimate degree of nanoparticle orientation and its intercalation/exfoliation state is mainly affected by the effectiveness of the solid state pre-dispersion of the nanoplatelets by means of ball-milling during the production of the ready-to-use formulation. The coating application and drying methods are additional factors, with a less important contribution allowing the achievement of similar oxygen permeabilities (*Q*_100_) after a single native protein coating in semi-industrial-scale vs. a triple pre-denatured coating at pilot scale.

In addition to the easy handling during processing of the studied ready-to-use formulations, their usage offers various additional advantages for converters, i.e., the possibility to use native, low viscosity WPI-based dispersions, which enable the handling of high solid content formulations (up to ~25% (*w*/*w*) ready-to-use formulation); their effective drying using patented process conditions; as well as a reduced dustiness of the ready-to-use formulations, leading to a greater nano-safety for operators by allowing the handling of agglomerated nanocomposite powder instead of free nanoparticles [38].
